# Current Trends, Innovations and Challenges in Non-Soy Tofu Production: A Critical Analysis

**DOI:** 10.3390/molecules31214032

**Published:** 2026-11-09

**Authors:** Patryk Siczek, Katarzyna Skrzypczak

**Affiliations:** Department of Plant Food Technology and Gastronomy, Faculty of Food Science and Biotechnology, University of Life Sciences in Lublin, Skromna 8, 20-704 Lublin, Poland; katarzyna.skrzypczak@up.edu.pl

**Keywords:** non-soy tofu, legumes, plant proteins, coagulation, gelation, texture, nutritional quality

## Abstract

Growing demand for diversified plant-based protein foods has stimulated interest in legumes as alternative raw materials for tofu production; however, knowledge on non-soy tofu remains fragmented across different species and processing systems. This review critically evaluates current knowledge on tofu and tofu-type products derived from non-soy legumes, linking raw-material composition and protein functionality with processing conditions and final product quality. Evidence on protein extraction, heat-induced transformations, coagulation, gel formation, texture, nutritional properties, and sensory quality was critically synthesised. The available studies demonstrate that successful curd formation depends not only on total protein content but also on storage-protein composition, non-protein constituents, and extraction, heating, and coagulation conditions. Consequently, processing conditions established for conventional soy tofu cannot be directly transferred across legume species. Emerging physical, enzymatic, and fermentation-based strategies offer additional opportunities to modify protein functionality and product structure, but their application in complete tofu systems remains insufficiently validated. Substantial methodological heterogeneity and limited direct interspecies comparisons currently restrict generalisation of processing recommendations. Future research should prioritise standardised comparative studies and integrated optimisation of technological, nutritional, sensory, and storage quality to support the development of reproducible and consumer-acceptable non-soy tofu products.

## 1. Introduction

The growing interest in plant-based foods is leading to an increase in demand for alternative sources of protein with high nutritional value and favourable processing properties. Legumes are particularly important, as they provide not only protein but also starch, dietary fibre, minerals and bioactive compounds. Soy remains the most popular source of plant-based protein with well-established technologies for processing. Its dominant role, however, is challenged by factors such as the allergenic potential of soy proteins and consumer concerns regarding genetically modified crops [1,2]. Consequently, the development of novel products based on other legumes may diversify plant protein sources and expand the range of soy-free alternatives available to consumers. Increasing research attention is directed towards peas (*Pisum sativum* L.), chickpeas (*Cicer arietinum* L.), faba beans (*Vicia faba* L.), mung beans (*Vigna radiata* L.), cowpeas (*Vigna ungiculata* L.) and other legume species. The use of these plants can increase the diversity of protein sources and the importance of raw materials adapted to local production conditions [3,4]. The significant growth in plant-based foods is linked to the need to improve the technological, nutritional and sensory quality of newly developed products; however, this is not simply a matter of switching from animal-derived raw materials or replacing soy with other legumes [5].

One of the main applications of these plants is the production of tofu and tofu-type products. The conventional process involves the extraction of seed components into an aqueous phase, heat treatment and protein coagulation, leading to the formation of a hydrated gel network. From a technological perspective, replacing soy with other legumes is not a simple raw-material substitution. Individual species differ in their protein content and fractional composition, the proportion of non-protein components, particularly starch, as well as protein solubility and the course of their denaturation, aggregation and gelation. These factors determine the efficiency of protein extraction, susceptibility to coagulation and the ability to form a three-dimensional water-retaining network, which in turn influences the yield, microstructure and texture of a tofu-type product [4,6,7,8,9,10,11].

The development of non-soy tofu requires consideration not only of the ability to form a stable curd, but also of process efficiency, water-holding capacity, texture, nutritional value as well as sensory quality. Particular challenges are posed by characteristic bean-like notes, bitterness, earthiness and textural differences, which may limit consumer acceptance. There remains a lack of research enabling a direct comparison of different legume species under identical processing conditions [3].

Previous reviews provide important but complementary perspectives on legume processing, tofu technology and the application of legumes in related structured plant-based foods, including dairy-free cheese alternatives [12]. Liu et al. [4] compared processing strategies for non-soy legumes and soybean, whereas reviews of tofu technology have focused on coagulants and coagulation mechanisms [13], processing and gelation [14], or broader aspects of raw materials, processing conditions and product development [15,16]. More recently, Sakhawat et al. [17] systematically reviewed legume-based tofu with particular emphasis on nutritional composition, physicochemical properties, sensory attributes, shelf life and storability. Collectively, these reviews provide substantial information on either general tofu technology or the quality attributes of alternative legume-based products, but they leave scope for a more integrated analysis linking species-specific raw-material characteristics and protein functionality with the successive processing operations responsible for non-soy curd formation.

Therefore, the present review adopts a process–structure–property perspective to critically analyse non-soy tofu and tofu-type products. Rather than considering individual product characteristics or processing variables independently, it examines how protein composition and non-protein matrix components interact with raw-material preparation, extraction, thermal treatment, coagulation and subsequent structure formation, and how these interactions determine the properties of the final product. Particular attention is given to distinguishing intrinsic differences between legume species from effects arising from heterogeneous experimental protocols and to evaluating where mechanistic evidence obtained from isolated proteins or model-gel systems can, and cannot, be extrapolated to complete tofu-type matrices. By integrating these relationships with evidence on physicochemical, nutritional and sensory quality, this review aims to identify methodological limitations, unresolved questions and priorities for the development of reproducible non-soy tofu technologies.

## 2. Literature Review Methodology

The literature search aimed to identify and evaluate studies on the use of non-soy legumes in the production of tofu and tofu-type products, including current trends, innovations, and key challenges. The literature search was conducted between June and August 2026 using Scopus, Web of Science, PubMed and ScienceDirect, with Google Scholar used as an additional source. Searches were performed in English and Polish and were restricted to articles and review papers. Where supported by the database interface, searches were conducted within title, abstract and keyword fields. Reference lists of relevant publications were additionally screened to identify potentially eligible studies not retrieved through the database searches. Moreover, the references lists of relevant articles were reviewed to identify further publications on the subject. The analysis covered both studies on the production of tofu from non-soy legumes and works describing the properties of legume proteins relevant to extraction, denaturation, aggregation, coagulation, gelation and the quality of the final product.

For the purposes of this review, the terminology used to describe legume-based products was interpreted according to the processing pathway and the physical form of the product rather than treated as interchangeable. The term “tofu” is used for products obtained from a legume-derived aqueous extract through protein coagulation and subsequent curd formation, with pressing applied where appropriate. “Tofu-type product” is used more broadly for non-soy products designed to resemble tofu but produced using modified processing routes or additional structure-forming components. The terms “curd” and “tofu-like gel” are retained when they reflect the terminology or product form reported in the original study. Thermally induced protein gels and other model-gel systems are treated as mechanistic evidence rather than as technologically equivalent to complete tofu products. Similarly, soy–legume blended tofu, fermented curds and dried tofu-type products are considered distinct product categories and are not directly equated with tofu produced exclusively from non-soy legumes. This distinction was applied when comparing processing conditions, texture, yield, water retention and other product characteristics across studies.

The search strategy was structured around three concept groups: (i) product-related terms (“non-soy tofu”, “legume tofu”, “tofu-type product”); (ii) raw-material terms (“chickpea”, “pea”, “faba bean”, “broad bean”, “lentil”, “lupin”, “mung bean”, “cowpea”, “Bambara groundnut”, “adzuki bean”, “kidney bean”, “pinto bean”); and (iii) processing and product-property terms (“protein extraction”, “heat treatment”, “coagulation”, “gelation”, “protein aggregation”, “coagulant”, “texture”, “rheology”, “microstructure”, “water-holding capacity”, “sensory evaluation”, “digestibility”, “phytate” and “antinutritional factors”). Terms from these groups were combined using the Boolean operators AND and OR and adapted to the syntax of the individual databases. Because the original database search histories were not retained, the exact database-specific search strings could not be reproduced retrospectively; the terms reported above therefore describe the search framework applied during the literature search rather than verbatim archived search queries. Bibliographic records were collected and organised using Zotero. The initial search identified 188 records. After removal of 13 duplicates, 175 unique records were screened on the basis of titles and abstracts. At this stage, 49 records were excluded because they did not meet the general scope of the review, leaving 126 publications for full-text assessment. Following full-text evaluation, 20 publications were excluded, primarily because they focused exclusively on soy or did not provide evidence relevant to the scope of the review. A total of 106 publications were retained for the qualitative synthesis. The final evidence base included peer-reviewed original studies on non-soy tofu production and related protein functionality, together with selected review articles and studies on soy systems used specifically to support the interpretation of coagulation and gelation mechanisms. Publications were eligible for inclusion if they addressed at least one of the following: (i) production or characterisation of tofu or tofu-type products prepared wholly or partly from non-soy legumes; (ii) extraction, thermal denaturation, aggregation, coagulation or gelation of legume proteins where the findings were relevant to understanding tofu structure formation; or (iii) physicochemical, nutritional, sensory or storage properties relevant to the evaluation of non-soy tofu-type products. Studies using isolated proteins or model-gel systems were retained as mechanistic evidence but were distinguished from studies evaluating complete tofu-type matrices. Selected studies on conventional soy tofu were included only when required to provide a mechanistic or technological reference for processes insufficiently characterised in non-soy systems.

The database searches were restricted primarily to articles and review papers published in English or Polish. Additional sources identified through Google Scholar or reference-list screening were considered when they provided directly relevant evidence not adequately represented in the database-derived literature. Publications were excluded when they fell outside the technological or compositional scope of the review, focused exclusively on soy without providing information required for mechanistic comparison, or did not provide sufficient information relevant to the production, structure or quality of tofu-type products. No publication-date restriction was applied, allowing older foundational studies to be retained while priority was given to recent evidence on emerging technologies and current research trends.

**Figure 1:**
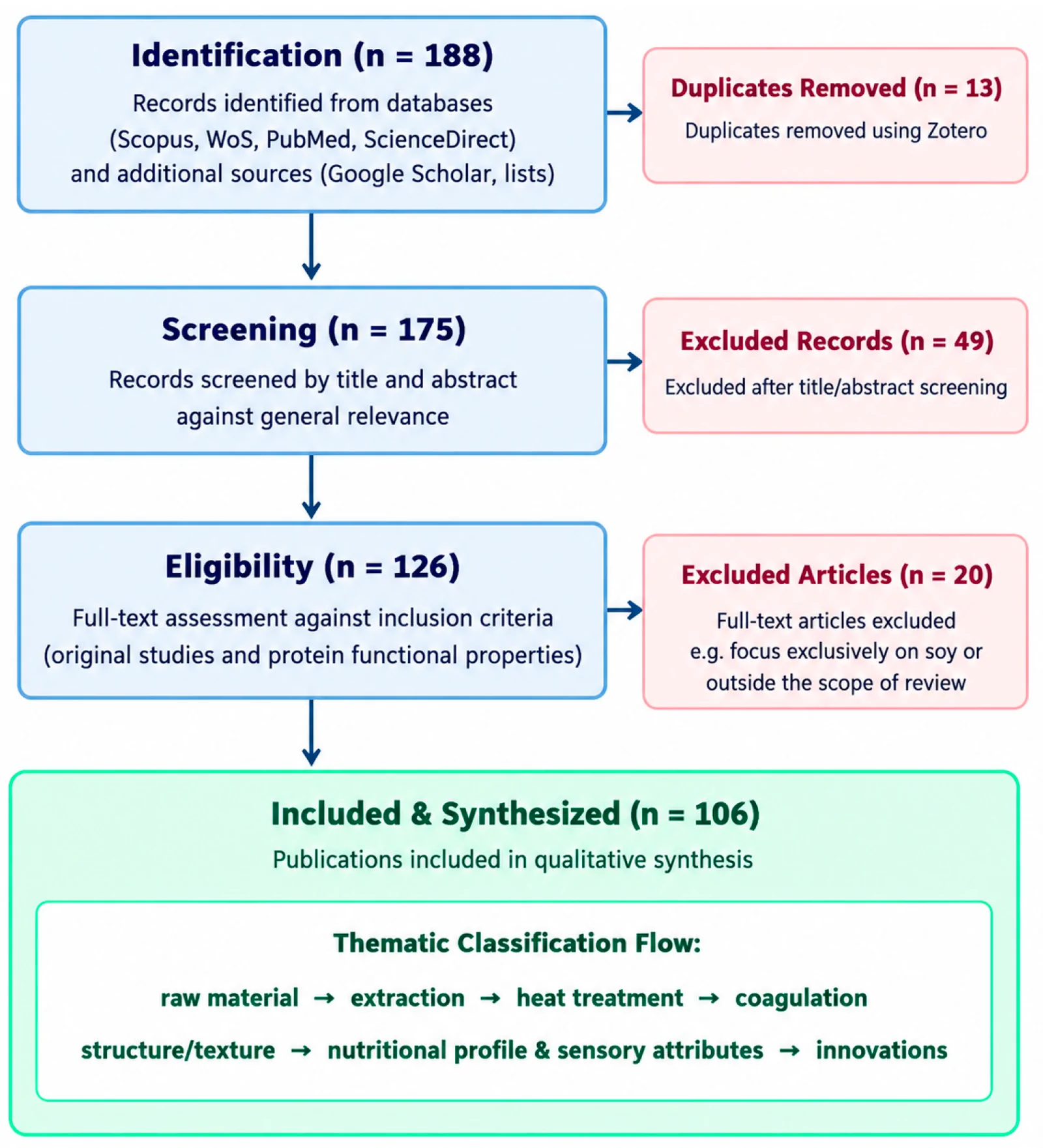
Flow diagram of the literature search and study selection process. Prepared by the authors, figure generated with the assistance of Google NotebookLM.

Due to the wide variation in raw materials, extraction methods, heat treatment conditions, the type and concentration of coagulants, and methods for assessing product properties, no meta-analysis was carried out. The results were subjected to a critical qualitative synthesis and comparative analysis, focusing on the relationships between raw-material properties, protein behaviour during processing, coagulation and gel-network formation, microstructure, and the quality of the final product. The literature search and study selection process is presented in Figure 1.

## 3. The Potential of Legumes as Raw Materials for the Production of Non-Soy Tofu

### 3.1. Composition and Characteristics of Legume Proteins

The storage proteins in legume seeds consist primarily of globulins, accompanied by smaller amounts of albumins [18]. Within the globulins, the 7S (vicillin) and 11S (legumins) fractions are of particular technological importance; their proportion, structure and properties vary between species [10,18]. Variations in the fractional composition and physicochemical properties of the proteins influence their solubility, thermal stability, aggregation and gel-forming ability [19,20].

Extensive comparisons of legume proteins show that the proportion and properties of globulin fractions may vary between species as well as between varieties, making it difficult to predict behaviour based solely on species [21]. Studies involving interspecies comparisons should take into account not only the total protein content but also the proportions of the storage fractions.

Studies investigating legumes such as peas, lentils, lupins and others reveal differences in the composition and properties of individual fractions [22,23,24,25,26], suggesting that the raw material should be assessed at the level of legumin, vicylin and albumin fractions, rather than solely on the basis of total protein content.

Cai et al. [7] demonstrated the technological significance of species-specific differences in protein composition by comparing curds prepared from soybean, chickpea, faba bean, pea, mung bean and lentil. Soybean, chickpea and faba bean exhibited a higher proportion of 11S globulins and produced harder curds (6.0–9.4 N) than pea, mung bean and lentil (4.2–4.9 N). Cohesiveness followed a similar pattern, reaching 0.67–0.77 in the former group and 0.57–0.59 in the latter [7]. Although these results indicate an association between storage-protein composition and curd texture, the higher proportion of 11S proteins should not be interpreted as a universal predictor of gel strength. Protein concentration, subunit composition, extraction method, thermal history, pH, ionic conditions and interactions with non-protein matrix components may also influence aggregation and network formation [10,11]. Therefore, differences in 7S/11S composition provide one explanation for species-specific curd properties, but their technological significance must be interpreted together with processing conditions and the composition of the complete legume matrix. Differences between protein fractions encompass not only their quantitative composition but also their structure and physicochemical properties [18]. Vicillin and legumins differ in this respect, which influences the formation of the protein network during gelation [10]. For this reason, the total protein content of the seeds is not a sufficient criterion for assessing their suitability for tofu production. The characteristics of the proteins involved in the formation of gel network are also crucial.

More recent studies of model tofu-type gels confirm the importance of the protein origin. Chen et al. [27], when comparing soy, chickpea and mung bean protein isolates coagulated with citric acid, demonstrated differences in the aggregation process as well as the properties of the resulting gels. These results indicate that the response to specific coagulation conditions depends on the protein source and should not be automatically extrapolated from soy to other species [27].

Legume seeds exhibit substantial variation in total protein content, overall composition, and the relative contributions of major storage-protein fractions. Differences in starch, lipid, and dietary fibre contents, as well as in the proportions of globulin fractions, influence both the nutritional properties of the raw material and its technological performance in tofu production (Table 1).

The comparison presented in Table 1 demonstrates that total protein content alone does not adequately describe the technological or nutritional potential of a legume raw material. Species with comparable protein contents may differ considerably in the relative contribution of their major storage-protein fractions, the proportions of non-protein components and their phytochemical profiles. These differences may contribute to species-specific behaviour during protein extraction, heat-induced denaturation, aggregation and coagulation and should therefore be considered when selecting raw materials for tofu-type products.

### 3.2. Protein Properties Important for Curd Formation in Tofu Production

In tofu technology, the solubility of proteins and their ability to denature, aggregate and form gels are of crucial importance. These properties depend both on the origin of the protein and on environmental parameters such as pH, temperature and ionic strength [46,47].

Solubility determines the amount of protein that can be present in the extract intended for coagulation. This characteristic strongly depends on pH and usually decreases in the range corresponding to the isoelectric point, whilst it increases at pH values further away from this point [48]. Therefore, the composition of the extract obtained from a given raw material is determined by both the initial protein content and the conditions applied during extraction.

The gelation of pea proteins is also influenced by the extraction method, fraction heterogeneity, pH, ionic strength and heating rate; therefore, gelling properties should be regarded as the result of interactions between the raw material and the process conditions [21].

Also, gelling functionality is determined by the applied method of isolation and the extent to which the native protein structure is preserved. Milder fractionation methods (dry fractionation or mild aqueous extraction that limit extensive pH shifts and protein denaturation) may preserve greater protein solubility and result in a different aggregation profile compared with conventional isoelectric precipitation, which affects the structure and strength of the gels [11,31,49]. Comparisons of peas, chickpeas and lentils, as well as studies on pea and chickpea isolates, indicate that solubility, water-binding capacity and behaviour during gelation may vary depending on the fractionation method and extraction conditions [31,48,50,51].

Significant differences between species also include the thermal properties of proteins. Withana-Gamage et al. [47] demonstrated variations in the functional and thermal properties of chickpea protein isolates, emphasising the importance of variety and the characteristics of the protein material for the technological stability of the isolates. In turn, Cai et al. [7], when comparing six legume species, indicated differences in the fractional composition of proteins, their thermal characteristics and the textural properties of the resulting curds. The results of these studies suggest that the heat treatment conditions preceding coagulation should be adapted to the properties of the specific raw material, rather than directly transferred from soy tofu technology [7].

Protein functionality can also be modified by the presence of other macromolecules. Jao et al. [52], in their study of lentil tofu, indicated that the use of CaSO_4_ and chitosan altered the proportion of major storage proteins in the soluble fraction and the textural properties of the product. In the case of CaSO_4_, aggregation of vicillin and legumin subunits into the insoluble fraction was observed, accompanied by an increase in the hardness and cohesiveness of the tofu. These results demonstrate that protein properties manifest themselves in interaction with the coagulation conditions applied, rather than solely as a characteristic of the raw material itself. Importantly, evidence obtained from isolated protein systems should be interpreted primarily as mechanistic evidence and should not be directly extrapolated to whole-legume tofu matrices, in which starch, fibre, lipids and other non-protein components may modify protein aggregation and water retention. Moreover, the functionality of proteins can also be deliberately modified enzymatically. In the case of mung bean protein, transglutaminase increased the degree of cross-linking, leading to a more compact and homogeneous microstructure and improvements in rheological, textural and water-holding capacity parameters [53,54]. Wang et al. [53] obtained maximum values for the storage modulus, hardness, elasticity, cohesiveness and water-holding capacity (WHC) (87%) at 30 U/g transglutaminase (TGase), whilst Vijayan et al. [54] demonstrated that at 2 IU/g and 3 h’ incubation, WHC increased from 1.24 to 3.77 g/g. These results indicate that the gelling capacity of non-soy proteins can be modulated after extraction and should not be regarded solely as a species-specific trait.

### 3.3. The Importance of Non-Protein Components

Non-protein components should be considered an integral part of the technological behaviour of non-soy legume matrices rather than merely a compositional difference between raw materials. In particular, many legumes investigated for tofu-type products, including peas, chickpeas, lentils, mung beans and faba beans, are starch-rich raw materials [4,10,20]. Consequently, during the heating of whole-seed extracts, protein denaturation occurs simultaneously with starch gelatinisation, while fibre and other polysaccharides may further modify network organisation, rheological behaviour and water retention [55,56,57]. The resulting structure therefore reflects interactions between proteins and the surrounding seed matrix rather than the behaviour of the protein fraction alone.

The technological importance of this matrix effect is particularly relevant when comparing studies based on different forms of raw material. Tofu-type products prepared from whole seeds or flours [44,58,59] retain starch, fibre, lipids and other seed constituents, whereas systems based on isolated proteins, such as that investigated by Chen et al. [27], substantially reduce the contribution of these components. Consequently, differences in gel strength, water retention or texture reported between studies cannot necessarily be attributed to intrinsic differences in protein functionality alone. The form of the starting material and the proportion of non-protein constituents should therefore be considered when comparing the technological suitability of different legumes for tofu production.

The technological relevance of non-protein components is also demonstrated by studies in which polysaccharides were deliberately introduced into tofu-type systems. In lentil tofu, Jao et al. [52] showed that chitosan modified protein aggregation and microstructure; increasing its concentration to 1% resulted in a more uneven, porous and brittle structure and was accompanied by deterioration of textural properties. In Bambara groundnut tofu, Chipeta et al. [43] demonstrated that gum arabic and sodium alginate modified textural and rheological properties, with the effects depending on the combination and proportion of the hydrocolloids. These findings show that polysaccharides do not act merely as inert constituents of the matrix but can alter protein network organisation and, consequently, the macroscopic properties of tofu-type products. Importantly, their effects are not necessarily beneficial: depending on their concentration and interactions with proteins and water, polysaccharides may either support structural development or disrupt the formation of a cohesive gel network [43,52].

The contribution of the native carbohydrate fraction should also be considered when interpreting the behaviour of whole-seed systems. Mung bean, for example, contains a substantial starch fraction with characteristic pasting behaviour and structural changes during heating [55]. Thus, differences between whole-seed tofu and gels prepared from isolated proteins may partly reflect the simultaneous transformations of proteins and starch rather than differences in protein functionality alone. This further demonstrates why results obtained using purified protein systems should be interpreted cautiously when predicting the behaviour of complete legume extracts.

### 3.4. Potential of Different Legume Species for the Production of Tofu-like Products

The available evidence indicates substantial species-specific differences in the ability of legume proteins to form tofu-type gels. Under standardised curd preparation conditions, Cai et al. [7] reported hardness values of 6.0–9.4 N for soybean, chickpea and faba bean curds, compared with 4.2–4.9 N for pea, mung bean and lentil curds. Cohesiveness followed a similar pattern, reaching 0.67–0.77 in the former group and 0.57–0.59 in the latter. These differences are technologically important because they demonstrate that the use of a protein-rich legume does not necessarily result in a curd with properties comparable to conventional tofu. Rather, curd formation reflects species-specific protein composition and functionality together with the response of the proteins to extraction, heating and coagulation conditions [7]. Consequently, differences observed between legume species should not be interpreted solely as evidence of their intrinsic suitability or unsuitability for tofu production, because processing conditions may either accentuate or compensate for differences in raw-material functionality.

Among the legumes compared by Cai et al. [7], chickpea showed particularly favourable curd-forming properties, which were associated with its storage-protein composition and, specifically, a relatively high contribution of 11S globulins. However, the technological potential of chickpea cannot be explained by protein composition alone. Studies on chickpea proteins indicate that gel formation is also influenced by the form of the starting material, protein concentration and processing conditions [29]. Moreover, Chen et al. [27], comparing citric-acid-coagulated protein isolates from soybean, chickpea and mung bean, demonstrated species-dependent differences in protein aggregation and the organisation of the resulting structures. Taken together, these findings suggest that the favourable curd-forming potential of chickpea is conditional on the processing system used and should not be interpreted as evidence that coagulation conditions developed for conventional soy tofu can be transferred directly to chickpea-based products [7,27,29].

In faba bean (*Vicia faba* L.), favourable curd-forming properties appear to result from the combined contribution of proteins and non-protein components. Jiang et al. [34] demonstrated that modification of the starch fraction affected gel strength and water-holding capacity, indicating that the behaviour of whole-legume systems cannot be predicted solely from protein composition. This technological potential should also be considered together with nutritional and sensory outcomes. Auer et al. [3] showed that tofu-type processing increased protein concentration and resulted in a higher degree of protein hydrolysis than cooking and fermentation. However, phytate was concentrated together with minerals, without a corresponding improvement in the estimated bioavailability of iron and zinc. Thus, increased protein or mineral concentration does not necessarily translate into improved overall nutritional quality. From a consumer perspective, Fernandez et al. [60] reported that faba bean tofu achieved the second-highest overall liking among the products tested, although earthy, musty and bitter sensory notes remained important limitations. Collectively, these findings illustrate a broader principle relevant to non-soy tofu development: favourable gel-forming behaviour alone is insufficient to establish the practical suitability of a legume, which must instead be evaluated simultaneously in terms of technological performance, nutritional consequences and sensory acceptance.

Mung bean provides a clear example of how processing conditions can modify the properties of a tofu-type product. Minh [59] showed that the raw-material-to-water ratio, heating time and temperature, and CaSO_4_·2H_2_O concentration influenced the structural and sensory characteristics of mung bean tofu. Among the conditions tested, a bean-to-water ratio of 1:5, heating for 15 min at 90 °C and 3% CaSO_4_·2H_2_O produced the most favourable results. The coagulation system itself may also influence product properties. Elygio et al. [61] demonstrated that the addition of whey from soy tofu production to the coagulation system affected the water content and hardness of mung bean curd. These findings indicate that the technological performance of mung bean cannot be considered independently of the processing protocol and further illustrate why direct comparisons between legume species are difficult when different extraction, heating and coagulation conditions are used.

Pea (*Pisum sativum* L.) provides another example of the strong dependence of tofu-type product properties on processing conditions. Under the standardised conditions applied by Cai et al. [7], pea curd exhibited lower hardness than soybean, chickpea and faba bean curds. However, this result should not be interpreted as evidence of inherently poor suitability of pea for tofu-type products. Studies on pea proteins and pea-based tofu-type systems demonstrate that their structural properties can be modified through appropriate process control [9,11], while Auer et al. [3] confirmed the feasibility of producing tofu-type products from both yellow and grey peas. Collectively, these findings indicate that the technological potential of pea depends on the interaction between raw-material characteristics and processing conditions rather than on protein content alone. They also demonstrate that conclusions regarding the relative suitability of individual legume species may be strongly influenced by the processing protocol used in a particular study.

Lentil provides a particularly clear example of why the technological potential of a legume should not be inferred from a single processing protocol. Under the standardised conditions used by Cai et al. [7], lentil curd exhibited comparatively low hardness and cohesiveness. In contrast, Jao et al. [52] demonstrated that the properties of lentil tofu could be substantially modified through the coagulation system. CaSO_4_ promoted aggregation of the major storage proteins and strengthened the resulting structure, whereas increasing chitosan concentration led to a less uniform, more porous and brittle network. Thus, the relatively poor performance of lentil under one set of processing conditions should not be equated with inherently low suitability of the species for tofu-type product development. Rather, the contrasting results emphasise the importance of matching coagulation conditions to the functional characteristics of the protein system.

Lupin (*Lupinus* spp.) represents a potentially valuable protein source, although the available evidence does not yet permit a robust assessment of its suitability for producing entirely lupin-based tofu. Most studies have examined lupin as a partial substitute for soybean rather than as the sole raw material. Jayasena et al. [62] reported that replacing up to 40% of soybean with lupin allowed acceptable tofu to be produced without substantial deterioration in texture, flavour or overall acceptability, although tofu yield decreased as the proportion of lupin increased. In a subsequent study using a 70:30 soy–lupin flour mixture, Jayasena et al. [63] demonstrated that both coagulant type and concentration affected yield and textural properties, further confirming that product quality depends on the interaction between raw-material composition and processing conditions. However, these findings should not be extrapolated directly to 100% lupin systems, because the presence of soy protein may substantially influence coagulation and network formation. Consequently, the limited evidence for soy-free, lupin-based tofu remains an important research gap.

Evidence regarding the suitability of cowpea (*Vigna unguiculata* L.) for tofu-type products remains limited. Manuel [64] evaluated products prepared from 100% chickpea, a 1:1 chickpea–cowpea mixture and 100% cowpea. The 100% cowpea formulation was rated as “very acceptable”, while the 1:1 chickpea–cowpea formulation achieved higher sensory acceptance. The characteristic bean aroma was identified as a limitation, and blanching before grinding was recommended to reduce its intensity. However, these findings provide only preliminary evidence for the suitability of cowpea for tofu-type applications. The products were formulated with added potato starch and were structured by heating and subsequent cooling rather than by conventional protein coagulation. Consequently, their sensory acceptability cannot be directly extrapolated to conventionally coagulated cowpea tofu. Systematic studies of cowpea protein coagulation, instrumental texture, microstructure and product stability are therefore required before its technological suitability for tofu production can be reliably assessed.

Bambara groundnut (*Vigna subterranea*) represents a different stage of development among the less-studied legumes, as recent research has progressed from demonstrating product feasibility towards deliberate modification of product structure and sensory properties. Chipeta et al. [43] showed that coagulant conditions and the addition of gum arabic and sodium alginate affected the textural and rheological properties of Bambara groundnut tofu. In a subsequent study, Chipeta et al. [44] extended this approach to physicochemical properties and consumer acceptance, with the formulation containing 0.6% glucono-delta-lactone (GDL) and 0.5% each of gum arabic and sodium alginate achieving the highest overall acceptance among the tested formulations. Together, these studies indicate that Bambara groundnut is technically feasible as a raw material for tofu-type products, but its available evidence base remains narrow. Importantly, the progression from feasibility testing to structure and sensory optimisation illustrates how research on alternative legumes can move beyond the simple replication of conventional tofu processing towards raw-material-specific product design.

Taken together, the available evidence does not support an unambiguous ranking of legume species according to their suitability for tofu production. Although some species show favourable structure-forming properties under particular experimental conditions, differences in raw-material preparation, extraction method, protein concentration, heating regime, coagulant type and concentration, and product formulation frequently confound species effects with processing effects. Consequently, a weak or strong gel reported in an individual study cannot be attributed solely to the intrinsic properties of the legume. This methodological heterogeneity also limits direct comparison of reported yield, texture and water-holding capacity across studies. Future comparative studies should therefore evaluate multiple legume species using standardised processing conditions and analytical methods, while subsequently optimising processing parameters for each raw material individually [7]. Such an approach would help distinguish intrinsic species-specific functionality from effects caused by differences in processing and provide a more robust basis for selecting raw materials for non-soy tofu production. The key technological characteristics, suitability for tofu-type product formation and main limitations of the selected legumes are summarised in Table 2.

## 4. Production Technology for Non-Soy Tofu

### 4.1. Preparation of Raw Materials

Raw-material preparation determines the composition and concentration of the extract entering subsequent heating and coagulation stages and should therefore be regarded as an integral part of tofu-type product formation rather than merely a preliminary operation. In mung bean tofu, Minh [59] demonstrated that changing the raw-material-to-water ratio affected both structural and sensory properties, with a 1:5 ratio providing the most favourable results among the tested conditions. In contrast, Bambara groundnut tofu has been produced from ground material rather than through conventional processing of whole soaked seeds [43]. These approaches illustrate an important source of methodological variability: changes in raw-material form and water ratio modify the composition and concentration of the material subjected to subsequent processing. Consequently, differences in yield, texture or water retention between studies cannot be attributed solely to legume species or coagulant type when the initial preparation procedures also differ.

### 4.2. Protein Extraction and Preparation of the Legume-Based Extract

Available studies indicate considerable variability in plant-based extract preparation procedures. This has significant methodological implications, as comparing tofu produced from different species solely on the basis of final firmness or yield may be subject to the influence of earlier stages of the process. When analysing the results, extraction parameters should be treated as an integral part of the technology, rather than merely as a preparatory stage.

The composition of the legume-based extract, which is subsequently coagulated, depends on the conditions under which the raw material is prepared and filtered. In a study by Jao et al. [58], a lentil extract was obtained by grinding hydrated seeds and then separating the insoluble fraction by filtration. In the case of mung beans, altering the seed-to-water ratio resulted in significant differences in the structural properties of the final product [59].

Protein extraction should therefore be evaluated not only in terms of recovery but also in terms of the suitability of the resulting extract for subsequent coagulation. Conventional aqueous extraction retains a relatively complex matrix, whereas alkaline extraction followed by isoelectric precipitation, ultrafiltration or dry fractionation produces protein fractions that differ in purity, structural state and functionality [20,31,48]. Consequently, results obtained with isolated or fractionated proteins cannot necessarily be extrapolated directly to whole-seed extracts used for tofu production.

Physical and enzyme-assisted extraction methods may further modify this balance between protein recovery and functionality. Ultrasound-assisted extraction has been investigated in faba bean and other legume systems [68,69,70], while combined pulsed electric field and ultrasound treatment has been proposed for chickpea protein extraction [71]. Similarly, Yang et al. [72] reported total protein extraction yields of 66.2% and 80.8% using optimised aqueous and enzyme-assisted extraction of black bean, respectively, together with differences in the functional properties of the recovered proteins. These findings demonstrate that a higher extraction yield does not necessarily indicate greater suitability for tofu production if the treatment simultaneously alters the properties required for subsequent aggregation and gel formation.

From a tofu-processing perspective, the critical outcome of the extraction stage is therefore not maximum protein recovery alone, but the production of an extract with sufficient protein concentration and appropriate functionality for heat-induced aggregation and subsequent coagulation. Emerging extraction technologies may provide opportunities to improve this balance, but evidence obtained from isolated protein systems should be validated in complete tofu-type production processes before technological advantages can be assumed.

### 4.3. Heat Treatment and Protein Denaturation

Heating is directly linked to structural changes in proteins that precede gel formation. In traditional tofu production, thermal denaturation prepares the proteins for subsequent aggregation and coagulation; the type of coagulant and the process conditions then determine the organisation of the gel network and the properties of the product [13,73]. In non-soy systems, the importance of thermal conditions is confirmed by studies on chickpea, faba bean and lentil proteins, in which the course of denaturation and intermolecular interactions influenced the final stiffness and nature of the gel network [10].

The available studies indicate that heating requirements are species- and process-dependent rather than transferable between legume systems. In mung bean tofu, Minh [59] identified heating at 90 °C for 15 min as the most favourable among the tested conditions, whereas Jao et al. [52] applied thermal gelation at 85 °C for 1 h in a lentil system subsequently modified with CaSO_4_ or chitosan. Although these studies used different raw materials and experimental designs and therefore cannot be compared directly, they demonstrate that effective structure formation can occur under markedly different thermal regimes. Heating should therefore be considered in relation to the thermal behaviour of the specific protein system and the subsequent coagulation conditions, rather than treated as a fixed processing step transferable from conventional soy tofu.

Studies investigating the model protein systems of mung beans and faba beans confirm that thermal conditions, pH, ionic strength and the extraction method influence the course of aggregation and the properties of the gel [56,74]. Therefore, current research is moving towards a more precise understanding of how heating conditions interact with protein composition, extraction history and the coagulation environment in different legume systems. Future studies should also investigate whether emerging technologies, such as ultrasound, pulsed electric fields or high-pressure processing, can be integrated with controlled heating to improve protein functionality and gel formation in non-soy tofu systems.

### 4.4. Mechanisms of Coagulation and Gel Formation

Coagulation converts the heat-modified protein dispersion into a continuous curd network by reducing the forces that maintain protein solubility and promoting intermolecular association. The resulting structure depends not only on coagulant type and concentration but also on the preceding degree of protein denaturation, protein composition, pH, ionic strength and the presence of non-protein matrix components [10,11,13,73]. Consequently, coagulation conditions cannot be considered independently of the extraction and heating stages discussed above.

Ionic coagulation, particularly with calcium and magnesium salts, promotes aggregation by reducing electrostatic repulsion between protein molecules and facilitating intermolecular association. The response is nevertheless strongly matrix-dependent. For example, CaSO_4_ concentrations identified as favourable in mung bean tofu cannot be transferred directly to other legumes because the resulting texture depends on the specific protein system and preceding processing conditions [59]. More mechanistic evidence is provided by lentil tofu: Jao et al. [52] showed that CaSO_4_ promoted the formation of a more compact structure and increased hardness and cohesiveness. SDS-PAGE further indicated a reduction in soluble vicilin and acidic and basic legumin subunits following CaSO_4_ addition, supporting their participation in aggregation into the insoluble network. Thus, the effect of a mineral coagulant is determined by its interaction with the particular protein matrix rather than by salt concentration alone.

At the molecular level, gel formation involves a combination of electrostatic effects and intermolecular interactions, including hydrophobic interactions, hydrogen bonding and disulphide bonding. Evidence from conventional tofu provides a useful mechanistic reference [13,75,76], while studies of isolated legume proteins demonstrate that the relative contribution of these interactions varies with species, protein fraction and environmental conditions [10,11]. This variability helps explain why identical coagulation strategies can produce markedly different structures in different legumes and reinforces the need for raw-material-specific optimisation.

### 4.5. Selection of Coagulation Method

The selection of a coagulation system is a critical stage in non-soy tofu production because its effectiveness depends on the properties and concentration of the proteins, their degree of denaturation, pH, ionic strength and the presence of non-protein matrix components. Available studies demonstrate that conditions effective for one legume cannot be assumed to be optimal for another [4,6,7,10,11]. Importantly, a distinction should be made between agents that directly induce protein coagulation, such as mineral salts and acidifying agents, and structure-modifying treatments or additives that alter an already developing protein network, including enzymatic cross-linking and hydrocolloid or polysaccharide addition.

Among direct coagulation approaches, calcium and magnesium salts are the most extensively investigated. Their effectiveness depends on both dosage and the properties of the protein matrix, and studies across different legume systems demonstrate substantial variation in coagulation behaviour and resulting product properties [6,52]. Selection of a mineral coagulant should therefore involve optimisation for the specific raw material rather than transfer of a fixed dosage between legume systems.

Controlled acidification provides a mechanistically distinct coagulation strategy. Glucono-delta-lactone (GDL) gradually lowers pH towards the isoelectric region, thereby reducing protein solubility and promoting aggregation. Studies on Bambara groundnut tofu demonstrate that GDL-based systems can produce tofu-type structures whose physicochemical and textural properties depend on both the acidification conditions and the presence of additional structuring agents [43,44]. Fermentation can provide another route to acid-induced coagulation: Gholivand et al. [77] used kombucha containing a symbiotic culture of bacteria and yeasts (SCOBY) to acidify legume-based extracts and obtain fermented curds. These approaches illustrate that acid-induced gel formation depends not only on the final pH but also on the route and kinetics of acidification.

Enzymatic cross-linking and polysaccharide or hydrocolloid addition should be distinguished from direct salt- or acid-induced coagulation. Transglutaminase (TGase) promotes covalent cross-linking between protein molecules and has been shown to modify the network structure and water-holding capacity of mung bean protein gels [53,54]. Chitosan similarly modified the texture and microstructure of lentil- and pea-based systems [52,58], while gum arabic and sodium alginate affected the structural properties of Bambara groundnut tofu [43,44]. These treatments may therefore complement coagulation by modifying network development, but their technological role should not be interpreted as equivalent to that of conventional coagulants.

Direct quantitative comparison of coagulant concentrations reported across studies is additionally limited by inconsistent expression of dosage. Concentrations have been reported using different units, including %, mM and g/L, and the reference basis is not always clearly specified, for example, whether the value refers to the mass or volume of extract, protein or total formulation. Consequently, numerical coagulant dosages should not be compared directly unless their calculation basis and processing conditions are equivalent. Future studies should report coagulant concentration using clearly defined units and reference bases to facilitate meaningful comparison between legume systems.

Overall, the available evidence does not identify a universal coagulation system or dosage suitable for all non-soy raw materials. Mineral salts and controlled acidification directly induce protein aggregation through different mechanisms, whereas enzymatic cross-linking and polysaccharide or hydrocolloid addition primarily provide additional means of modifying network formation. Selection and optimisation should therefore be performed for each raw material while considering protein composition and concentration, thermal history, pH, ionic conditions and non-protein matrix components. Standardised reporting of these variables is essential for distinguishing genuine raw-material effects from differences caused by experimental design.

### 4.6. Forming, Pressing and Structure Formation

Coagulation does not necessarily represent the final stage of structure formation. In conventional tofu production, the resulting curd may be formed and pressed to remove part of the aqueous phase [43], whereas studies on non-soy systems apply substantially different post-coagulation procedures. Some investigate pressed products resembling conventional tofu, while others examine unpressed curds or thermally formed protein gels. These products should not be regarded as technologically equivalent because pressing alters moisture content, density and the organisation of the protein network. For example, Jao et al. [52] produced a lentil-based system by adding CaSO_4_ or chitosan before heating at 85 °C for 1 h; even the control without these additives was thermally gelled. The resulting material therefore represents a different structure-forming pathway from a process in which a coagulated curd is subsequently pressed. Such methodological differences must be considered when comparing texture, water retention or yield across studies.

Methodological differences have significant implications for the interpretation of the literature. Examples of processing conditions applied to different non-soy legumes and their main effects on product properties are summarised in Table 3. Results concerning hardness, water content or yield should not be compared without taking into account the method of extract preparation, heating conditions, type of coagulant and product-forming method. This is illustrated by the different effects observed for mung bean, lentil and *Vigna subterranea* products when different processing procedures were applied [43,52,59].

**Figure 2:**
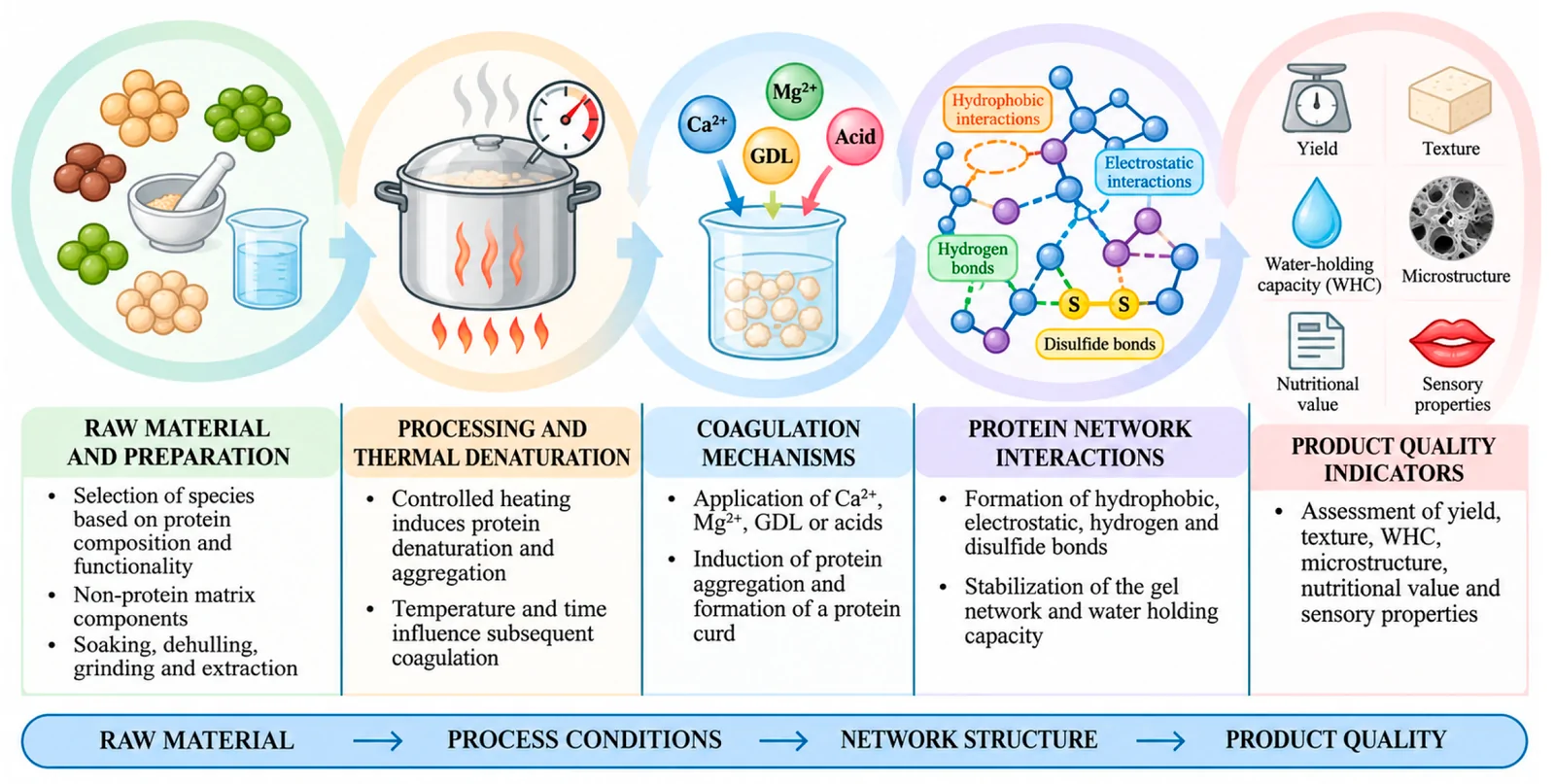
Factors determining the formation and quality of non-soy tofu. Source: Authors’ own concept; figure generated with the assistance of Google NotebookLM.

The available results suggest that the production of non-soy tofu should be regarded as a sequence of interrelated technological operations. Extract preparation parameters determine the properties of the material being heated; thermal conditions influence its potential for subsequent structure formation; and the type and dosage of the coagulant, as well as the product-forming method, shape the properties of the final product. Significant differences among the methods used currently limit the possibility of identifying universal process parameters applicable to all non-soy legumes. The main factors determining the formation and quality of non-soy tofu and their interrelationships are summarised in Figure 2.

## 5. Relationships Between Process Conditions and the Quality of Non-Soy Tofu

### 5.1. Product Yield and Water Retention

Reported tofu yield must be interpreted according to the basis on which it is calculated. Fresh-product yield reflects the mass of tofu obtained relative to the starting material and is therefore strongly influenced by product moisture, whereas dry-matter recovery and protein recovery quantify the proportion of solids or protein retained during processing and represent distinct measures of process efficiency. Water-holding capacity (WHC) is likewise a separate parameter describing the ability of the gel network to retain water under defined analytical conditions. Consequently, fresh yield, moisture content, dry-matter or protein recovery and WHC should not be used interchangeably, and high fresh-product yield should not automatically be regarded as an indicator of higher process efficiency or product quality. This relationship is illustrated by a study on mung bean curd, in which soy tofu whey was used as part of the coagulation system together with 1% citric acid [61]. Different concentrations of soy tofu whey (0%, 2%, 4%, 6% and 8%) significantly affected curd yield, moisture content, protein content and hardness. Among the tested variants, the addition of 2% whey was identified as the most favourable, providing the highest yield, protein content and hardness [61]. These findings demonstrate that the amount of coagulant used can simultaneously influence water retention, yield and the structural properties of mung bean curd.

The importance of water retention is also demonstrated by studies on tofu made from *Vigna subterranea*. Chipeta et al. [43] showed that the type of coagulant and the addition of gum arabic and sodium alginate influenced the physicochemical and textural properties of the product. The effects of hydrocolloids depended on their combination and proportion, suggesting that water retention within the matrix should be considered together with changes in the mechanical properties of the product.

Yield and water retention in tofu-type products should therefore be interpreted in relation to product texture and microstructure. Retaining a large amount of water may increase product weight but may also result in a weaker and more deformable structure, as illustrated by the findings for mung bean and Bambara groundnut products described above. Product yield and water retention should also be considered in relation to the type of raw material and coagulation method. Studies on tofu and tofu-type products indicate that changes in the coagulation system can simultaneously affect composition, texture and sensory properties [78,79,80]. Therefore, high yield alone should not be regarded as a sufficient criterion for process optimisation.

### 5.2. Microstructure and the Organisation of the Gel Network

Microstructural analysis allows the mechanical properties of tofu to be linked to the organisation of its matrix. In a well-formed curd, protein aggregates form a three-dimensional structure responsible for the mechanical properties of the product and the retention of the aqueous phase. However, the nature of this structure can vary significantly depending on the coagulant used and the composition of the system.

In lentil tofu, Jao et al. [52] demonstrated that the use of CaSO_4_ resulted in a more compact structure than in the thermally gelled control sample, which was associated with increased hardness and cohesiveness. Conversely, increasing the chitosan concentration to 1% resulted in a more uneven, porous and brittle structure, accompanied by a deterioration in textural properties. These observations demonstrate that changes in the organisation of the protein network are directly reflected in the macroscopic properties of the product.

Mechanistic evidence from isolated protein systems provides additional insight into species-dependent network formation, but should be distinguished from evidence obtained from complete tofu-type matrices. H.-L. Chen et al. [27] showed that, following citric acid treatment, chickpea protein isolate formed larger floccules without a clearly developed continuous network, whereas mung bean protein isolate formed a more irregular and porous structure. These findings demonstrate differences in the aggregation behaviour of the isolated proteins, but they should not be interpreted as direct evidence of the microstructure expected in whole-legume tofu, where starch, fibre, lipids, minerals and other matrix components may modify protein aggregation and water retention. Microstructural analysis is therefore particularly useful for interpreting the results of instrumental texture measurements. Hardness alone does not allow an unambiguous determination of whether a change in texture results from the formation of a more compact protein network, differences in water retention or the influence of other matrix components. An integrated analysis of microstructure, texture and water retention can therefore provide a more comprehensive characterisation of the quality of non-soy tofu.

A further example of the relationship between microstructure and functionality is provided by transglutaminase-crosslinked mung bean protein gels. Transglutaminase treatment promoted the transition from a loose and heterogeneous structure towards a denser network with smaller and more uniform pores, accompanied by improvements in gel properties, including hardness and water-holding capacity [53,54]. These findings provide mechanistic evidence of how enzymatic cross-linking can modify protein network organisation, but validation in complete mung bean tofu matrices is required before equivalent effects on tofu texture or water retention can be assumed.

More broadly, studies on the gelation and modification of legume proteins indicate that the mode of protein aggregation and the degree of network formation depend on both environmental conditions and the processing methods applied [49,53,74]. Microstructure therefore provides an important link between molecular changes in proteins and the macroscopic quality characteristics of tofu-type products.

### 5.3. Texture and Rheological Properties

Texture is one of the most frequently analysed characteristics of tofu and is a key parameter distinguishing products obtained from different raw materials. In a direct comparison of six legume species, Cai et al. [7] demonstrated that curds made from soybeans, chickpeas and faba beans achieved a hardness of 6.0–9.4 N and a cohesiveness of 0.67–0.77, whereas products made from peas, mung beans and lentils were characterised by a hardness of 4.2–4.9 N and a cohesiveness of 0.57–0.59. The higher proportion of 11S globulins in the first group was associated with more favourable textural properties; however, this relationship should not be interpreted as a universal causal effect, because curd texture is also influenced by protein concentration and subunit composition, thermal history, pH, ionic conditions, extraction method and interactions with non-protein matrix components [10,11].

The textural properties of the same raw material can be significantly modified by coagulation conditions. Jao et al. [52] demonstrated that the use of 12 mM CaSO_4_ increased the hardness and cohesiveness of lentil tofu compared with the thermally gelled control sample, whereas the addition of 1% chitosan reduced both parameters. These findings indicate that texture results from interactions between protein properties and coagulation conditions rather than solely from the type of raw material.

A similar relationship was observed for Bambara groundnut (*Vigna subterranea*), for which the concentration and type of coagulant significantly affected tofu hardness. Chipeta et al. [43] reported that increasing the GDL concentration from 0.3% to 1% increased hardness from 1.96 to 6.73 N. Tofu coagulated with 4% vinegar or 4% lemon juice showed hardness values of 4.14 and 3.04 N, respectively. These results demonstrate that even within the same raw material, the type and concentration of coagulant can substantially modify texture. Consequently, comparisons of tofu hardness between studies should account for coagulation conditions rather than attributing differences solely to the legume species.

Mechanistic studies using model protein gels further demonstrate that the response to processing conditions depends on the protein source. H.-L. Chen et al. [27] showed that the addition of 0.1% citric acid reduced the hardness of thermally induced gels prepared from soy, chickpea and mung bean protein isolates, although the magnitude of the changes and the structure of the resulting gels varied with the protein source. Because these experiments were conducted using isolated proteins rather than complete legume matrices, the results should be interpreted as evidence of species-dependent protein behaviour rather than as direct predictions of tofu texture. Nevertheless, they help explain why processing conditions optimised for one protein source cannot necessarily be transferred directly to another.

When comparing textural properties across studies, differences in raw materials, coagulants and preparation procedures should therefore be taken into account. These factors can lead to different relationships between hardness, cohesiveness, elasticity and sensory acceptance. Such relationships have been reported both in tofu produced from alternative raw materials and in studies comparing different coagulation systems [79,81,82].

### 5.4. Nutritional Value and Digestibility

One of the main objectives of using pulses in tofu production is to obtain a product with a high protein content. Coagulation allows some of the proteins originally present in the seeds to be concentrated in the hydrated curd. Auer et al. [3], when comparing different processing methods for faba beans, yellow peas, grey peas and soybeans, found that products obtained by protein coagulation had the highest protein content among the processing variants analysed. Interesting results have also been reported for chickpea tofu. Dimassi et al. [65] demonstrated that 59.1% of the initial protein content of the seeds was retained in the tofu itself, whilst a total of 75.1% of the protein was recovered from the tofu, okara and separated starch fraction combined. The resulting chickpea tofu contained 56.18% protein on a dry basis. The high protein retention efficiency partially compensated for the lower initial protein content of chickpeas compared with soybeans.

However, the composition of the product may depend strongly on the formulation. In tofu produced from mixtures of soybeans and chickpeas, increasing the proportion of chickpeas from 0% to 30% resulted in a gradual decrease in protein content from 12.26% to 10.22% and in fat content from 4.18% to 2.36%, whilst simultaneously increasing fibre content from 0.01% to 2.80% on a wet-weight basis [83]. These results relate to products obtained from blended raw materials rather than entirely soy-free tofu, but they demonstrate that replacing soy with another raw material can lead to simultaneous changes in several nutrients. The potential to produce high-protein products has also been demonstrated for *Vigna subterranea*. In dried tofu pieces, Chipeta et al. [44] reported a protein content exceeding 40%, although this value depended on the coagulants and hydrocolloids used. The resulting products were considered a potential high-protein alternative to textured soy products. It should be noted, however, that these results refer to dried products and therefore should not be directly compared with the protein content of fresh tofu.

An assessment of nutritional value requires a broader consideration of raw-material composition. Pulses differ in their amino acid profiles, mineral and fibre contents, and bioactive compounds, whilst processing may further modify the availability of these components. Legume seeds contain a range of bioactive compounds, including phenolic acids, flavonoids, tannins and saponins, although their composition and concentration vary considerably among species and cultivars [30,36,84]. These compounds may contribute to the antioxidant and other biological properties of legume-based foods, whilst their retention and availability can be modified during processing. Therefore, findings concerning tofu should be interpreted in relation to the characteristics of the raw material [85]. In tofu prepared with varying proportions of mung beans and soybeans, increasing the proportion of mung beans increased flavonoid content and 2,2-diphenyl-1-picrylhydrazyl (DPPH) radical-scavenging activity, whereas total phenolic content and 2,2′-azino-bis(3-ethylbenzothiazoline-6-sulfonic acid) (ABTS) activity did not differ significantly among the variants and α-glucosidase inhibitory activity was highest in pure soy tofu [86].

Protein content alone does not determine nutritional value; the availability of proteins to digestive enzymes is also important. Particularly valuable data in this regard were provided by Auer et al. [3], who used an in vitro digestion model to compare products made from faba beans, yellow peas, grey peas and soybeans. Coagulated tofu-type products exhibited the highest degree of protein hydrolysis among the processing methods analysed, indicating increased protein accessibility to digestive enzymes. The response nevertheless depended on the legume species and processing method, indicating that a single processing approach cannot be assumed to provide optimal nutritional properties for all pulses. These findings are relevant to non-soy tofu because they demonstrate that coagulation may not only enable product formation but also influence protein availability during digestion. However, these results should be interpreted cautiously because they were obtained using an in vitro model rather than human nutritional studies.

Legumes also contain compounds that may limit the utilisation of certain nutrients. In the context of tofu, phytate is particularly important because it can chelate minerals such as iron and zinc and thereby reduce their potential bioavailability. Importantly, tofu processing may concentrate protein and minerals without producing a corresponding reduction in phytate. Auer et al. [3] demonstrated that coagulation did not effectively remove phytate; consequently, the higher concentration of nutrients in tofu-type products did not translate into improved estimated iron and zinc bioavailability. In contrast, fermentation was substantially more effective in reducing phytate, with complete degradation of detectable phytate reported in fermented products prepared from faba beans and grey peas [3]. These findings demonstrate that increased protein or mineral concentration should not be interpreted as evidence of improved nutritional quality when compounds restricting mineral utilisation remain in the product. Data on chickpeas further indicate that soaking, germination and heat treatment can reduce phytate, protease inhibitors and other antinutritional factors, although their effectiveness depends on the treatment applied [87,88]. Optimisation of non-soy tofu processing should therefore consider phytate reduction alongside protein recovery, texture and yield rather than treating these technological outcomes independently of mineral bioavailability.

Processing can also affect both mineral content and potential mineral bioavailability. The type of coagulant is particularly relevant because calcium- or magnesium-containing salts can alter the content of the respective minerals in the final product. In chickpea tofu, Dimassi et al. [65] reported an ash content of 6.29% on a dry basis, which was associated with mineral uptake from the calcium coagulant used during processing. The study used CaCl_2_ as the coagulant at a concentration of 2.5 g/L. However, a high mineral content does not necessarily indicate high mineral bioavailability. Auer et al. [3] assessed the potential bioavailability of iron and zinc using molar ratios of phytic acid to minerals and additionally evaluated iron bioavailability using a Caco-2/HT29-MTX cell model and ferritin formation. Fermented products, characterised by lower phytate contents, showed more favourable indicators of potential mineral bioavailability than the corresponding tofu products. These findings point to a nutritional trade-off: coagulation may promote protein concentration and accessibility but does not necessarily maximise mineral bioavailability. More broadly, nutritional quality results from the interaction between legume species and processing method rather than from a single universally beneficial processing approach.

The available findings indicate that tofu made from alternative pulses can be a valuable source of protein; however, its nutritional quality should be assessed using multiple criteria. Studies on chickpeas demonstrate that a substantial proportion of seed protein can be retained in the resulting tofu [65], whilst comparative studies of faba beans and peas indicate that coagulation can enhance protein hydrolysis during in vitro digestion [3]. At the same time, the retention of phytates in tofu may limit the potential bioavailability of iron and zinc. Future studies should therefore combine assessment of basic composition with analyses of amino acid profiles, protein digestibility, antinutritional factors and mineral bioavailability. A significant limitation of the current literature remains the small number of comprehensive studies, particularly direct comparisons of multiple legume species conducted using the same protocol and studies confirming in vitro observations under in vivo conditions.

### 5.5. Food Safety, Allergenicity and Antinutrtrional Factors

The replacement of soybean with other legumes does not necessarily result in an allergen-free product [89]. Although non-soy tofu may provide an alternative for consumers who avoid soy, several legumes used for tofu-type products contain clinically relevant allergens, and immunological cross-reactivity may occur between different legume species. Lupin is a particularly important example because clinically relevant lupin allergy and cross-reactivity with other legumes have been documented [90]. Consequently, the development and labelling of non-soy tofu should consider the allergenic potential of the specific raw material rather than presenting the absence of soy as equivalent to the absence of legume allergens [90].

Food-safety assessment should also account for antinutritional factors and naturally occurring compounds whose relevance differs between legume species. Phytate can reduce the potential bioavailability of minerals, and, as discussed above, tofu-type processing may concentrate phytate together with protein and minerals rather than effectively removing it [3]. Legumes may additionally contain protease inhibitors and lectins, some of which can be substantially reduced or inactivated by appropriate soaking and thermal processing [87,88]. However, the effectiveness of these treatments depends on the raw material and processing conditions, and adequate heat treatment should therefore be validated for the specific legume and process rather than assumed from conventional soy tofu production.

Species-specific hazards require additional consideration. Faba bean contains the pyrimidine glycosides vicine and convicine, whose metabolites are associated with favism in susceptible individuals, particularly those with glucose-6-phosphate dehydrogenase deficiency [91]. Processing methods such as soaking and thermal treatment can reduce their concentrations, but the extent of reduction depends on the processing conditions [91]. Lupin presents a different concern because its seeds may contain quinolizidine alkaloids; their concentration varies with species and cultivar, and appropriate selection of low-alkaloid material or validated debittering procedures is therefore required when lupin is intended for food applications [92].

These considerations demonstrate that technological optimisation of non-soy tofu cannot be separated from safety assessment. Raw-material selection and pre-treatment should be designed not only to maximise protein extraction, coagulation and desirable texture but also to control relevant allergens, heat-labile antinutritional factors and species-specific toxicants. Future studies should therefore report the pre-treatment and heating conditions sufficiently clearly to permit assessment of their effectiveness in controlling these compounds and, where relevant, directly quantify their residual concentrations in the final tofu-type product.

### 5.6. Sensory Quality and Consumer Acceptance

Sensory acceptance is one of the main criteria determining whether non-soy pulses can be successfully used in tofu production. Even if a product has an appropriate yield, protein content and gel structure, its practical application may be limited if its flavour, aroma, texture or appearance do not meet consumer expectations. Available studies indicate that both the characteristic sensory attributes of pulses and the textural properties of the resulting product can influence consumer acceptance.

The characteristic aroma of legume seeds may reduce the sensory quality of plant-based products. Manuel [64], who investigated the potential of cowpea for tofu production using formulations based on chickpea and cowpea, identified the characteristic odour of cowpea as a factor affecting product aroma. This finding highlights the importance of controlling undesirable sensory attributes from the raw-material preparation stage. More broadly, studies on legume-based foods indicate that reducing beany and other undesirable flavour and aroma notes is important for improving sensory acceptance [67,93]. This is particularly relevant to non-soy tofu, for which differences in legume species, seed composition, pre-treatment and processing conditions may affect the intensity of characteristic sensory notes. Future studies should therefore assess not only overall acceptability but also the individual sensory attributes responsible for reduced consumer acceptance.

Instrumentally measured textural parameters, such as hardness and cohesiveness, are also relevant to the sensory perception of tofu. In a study on mung bean tofu, Minh [59] evaluated structural, colour and sensory properties simultaneously. The most favourable sensory characteristics were obtained at a bean-to-water ratio of 1:5, whilst subsequent optimisation identified heating at 90 °C for 15 min and the use of 3% CaSO_4_·2H_2_O as suitable processing conditions. These findings demonstrate that the optimisation of instrumental texture should be considered together with the sensory evaluation of the final product.

The relationship between formulation and sensory acceptance has also been demonstrated for Bambara groundnut (*Vigna subterranea*) tofu. Chipeta et al. [44] showed that the type of coagulant and the addition of hydrocolloids influenced consumer acceptance. The highest overall acceptability score, 3.92 on a five-point scale, was obtained for dried tofu pieces containing 0.6% GDL, 0.5% gum arabic and 0.5% sodium alginate. Products containing GDL alone and those containing vinegar combined with hydrocolloids received scores of 3.58 and 3.55, respectively. These findings demonstrate that technological modifications can affect consumer perception as well as the structural properties of the product. However, greater firmness should not automatically be interpreted as greater sensory acceptance, as the desired texture depends on the characteristics of the product and consumer expectations.

Studies in which non-soy legumes were used as partial substitutes for soybeans provide additional information on the sensory consequences of changing raw-material composition. Kumar and Premagowri [94] evaluated tofu formulations containing a mixture of chickpeas, mung beans and horse gram in combination with soy. Increasing the proportion of the non-soy legume mixture was associated with more pronounced off-flavours and a more crumbly and brittle texture. Although these products were not entirely soy-free, the findings demonstrate that the proportion of alternative legume ingredients can influence both flavour and texture. However, they should not be interpreted as evidence that partial soy substitution is generally more acceptable than completely soy-free tofu, as the observed effects were specific to the formulation and processing conditions investigated.

One of the main limitations of the current literature on non-soy tofu is the relatively small number of consumer studies compared with studies focusing on composition, gelation and instrumental texture. In addition, differences in rating scales, panel composition and size, sample preparation and evaluation procedures make direct comparisons between studies difficult. The available evidence nevertheless highlights the importance of combining instrumental characterisation with sensory analysis and consumer testing. Such an approach would make it possible to determine not only whether a technological modification improves parameters such as hardness or water-holding capacity, but also whether these changes result in a product that is acceptable to consumers. The relationships between selected raw materials and processing factors, changes in product microstructure and their resulting effects on the quality characteristics of non-soy tofu are summarised in Table 4.

## 6. Technological Challenges and Strategies for Overcoming Them

### 6.1. Limited Protein Extraction and Recovery

Limited protein recovery remains one of the main technological challenges in the development of non-soy tofu. Extraction efficiency affects not only the amount of protein transferred to the aqueous phase but also the functional properties of the recovered proteins, including those relevant to subsequent aggregation and gel formation. Extraction conditions should therefore be adapted to the characteristics of the specific raw material, with the aim of increasing protein recovery whilst limiting undesirable structural changes and preserving protein functionality [4,11].

Future research should focus on extraction strategies that combine high protein recovery with the preservation of functional properties. Their effectiveness should be evaluated not only in terms of laboratory-scale extraction efficiency but also with regard to energy consumption and scalability. Studies on peas, chickpeas, faba beans and black beans have investigated different approaches to protein recovery, including physical and enzymatic methods and their combinations [68,71,72,95]. These approaches may provide opportunities to improve protein recovery while maintaining the functionality required for subsequent gel formation. However, their suitability should ultimately be evaluated directly in complete non-soy tofu production systems.

### 6.2. Limited Coagulation Ability and Formation of a Stable Gel Network

Limited coagulation and gel-forming ability may result from differences in protein composition and functionality as well as from the presence of starch and other non-protein matrix components. Studies on non-soy curds and model legume protein systems indicate that gel formation can be modified by factors, including thermal conditions, pH, ionic strength and protein cross-linking [53,74,96]. Future research should therefore focus on determining how these factors interact in complete tofu systems rather than evaluating them independently.

### 6.3. Obtaining the Desired Texture and Water Retention

When comparing studies, yield, recovery, moisture content and water-holding capacity (WHC) should be treated as distinct parameters. Fresh tofu yield describes the mass of the obtained product relative to the amount of starting material and is therefore strongly influenced by the amount of water retained in the curd. In contrast, dry-matter recovery and protein recovery describe the proportion of the initial solids or protein, respectively, recovered in the final product or a specified process fraction. Moisture content indicates the proportion of water present in the product, whereas WHC describes the ability of the gel network to retain water under the conditions of the analytical method. Consequently, these parameters are not interchangeable, and numerical yield values reported by different studies should not be compared directly unless the calculation basis, product moisture and processing conditions are sufficiently comparable.

The available evidence indicates that yield, water retention, texture and microstructure are closely interrelated, but the relationships between these characteristics are not necessarily linear. Changes in coagulation conditions can simultaneously affect water retention and the mechanical properties of the resulting curd. For example, Chipeta et al. [43] demonstrated that the hardness of Bambara groundnut tofu depended on both the type and concentration of the coagulant. Similarly, studies on lentil- and pea-based tofu-type products showed that the effect of chitosan was concentration-dependent and that higher concentrations did not necessarily improve gel texture or network organisation [52,58].

Consequently, no single physicochemical parameter can be used to define optimal quality across all non-soy tofu products. Yield should be interpreted together with water retention, whilst instrumental texture should be considered in relation to microstructure and sensory quality. Moreover, the relationships among these characteristics may differ according to the legume species and the processing conditions applied [7,27,43].

The properties of tofu may also be modified by treatments applied to the protein system before coagulation. Physical processing methods, such as ultrasound, high-pressure homogenisation and pulsed electric fields (PEFs), can modify protein structure and thereby affect properties relevant to subsequent aggregation and gel formation. Enzymatic treatment with transglutaminase provides a different mechanism by promoting cross-linking between protein molecules. Such pre-treatments may therefore offer opportunities to modify the gel-forming behaviour and texture of legume protein systems, although their effects depend on the protein source and processing conditions and require further validation in complete tofu-type products [49,53,97,98].

### 6.4. Flavour, Aroma, Colour

The characteristic aroma of legume seeds constitutes a significant limitation to the sensory quality of plant-based products, including tofu. Manuel [64], investigating the potential of cowpea (*Vigna unguiculata*) for tofu production, identified the strong bean-like odour of cowpea as a factor affecting product aroma. Blanching the cowpeas before grinding was proposed as a potential means of reducing this undesirable odour. Given the limited scope of the study, these findings should be regarded as preliminary; nevertheless, they highlight the importance of controlling aroma from the raw-material preparation stage.

Undesirable aromas are a broader challenge in legume-based products. The literature on alternative plant-based foods indicates that reducing beany and other undesirable flavour and aroma notes is important for improving sensory acceptance [67,93]. In non-soy tofu, differences in raw-material composition, pre-treatment and processing conditions may influence the intensity of characteristic sensory notes. Future studies should therefore assess not only overall acceptability but also the specific sensory attributes responsible for reduced consumer acceptance.

The sensory profile is strongly dependent on the legume species and processing conditions. In faba beans, the formation of undesirable flavour and aroma compounds has been associated with lipid-derived volatile compounds, whereas lupins contain numerous aroma-active compounds that may contribute to their characteristic sensory profile. Consequently, strategies for reducing beany, grassy or bitter notes should be adapted to the specific raw material rather than regarded as a universal processing step [99,100,101]. Processing strategies such as fermentation, controlled heat treatment and other physical treatments may provide opportunities to modify undesirable sensory characteristics, although their effectiveness depends on the raw material and processing conditions [93].

Studies on mung bean tofu further indicate that changes in processing conditions may affect structural and sensory properties simultaneously, highlighting the importance of considering these characteristics together when optimising tofu-type products [59]. The importance of linking technological optimisation with sensory evaluation was also demonstrated for Bambara groundnut tofu. Chipeta et al. [44] found that formulations differing in coagulant and hydrocolloid content received different consumer ratings, with the formulation containing 0.6% GDL together with 0.5% gum arabic and 0.5% sodium alginate achieving the highest overall acceptability score. These findings demonstrate that modifications intended to improve product structure should also be evaluated in terms of their effects on consumer perception.

The sensory profile may also change when non-soy legumes are incorporated into soy-based tofu formulations. Kumar and Premagowri [94] observed that increasing the proportion of a chickpea, mung bean and horse gram mixture was associated with more pronounced off-flavours and a more crumbly and brittle texture. This finding further illustrates that the sensory consequences of incorporating alternative legumes depend on both their proportion in the formulation and the processing system used.

### 6.5. Antinutrients and Their Modifications During Processing

Pulses contain several compounds that may reduce nutrient utilisation, including phytates, protease inhibitors, lectins and tannins. Phytates are particularly relevant because they can bind minerals such as iron and zinc and thereby reduce their potential bioavailability. Their concentration and nutritional significance depend on both the legume species and the processing method applied. Soaking, heating, germination and fermentation can modify the levels of these compounds, although their effectiveness varies among processes and raw materials [3,102,103,104].

Processing may also affect mineral composition and bioavailability. The choice of coagulant is particularly relevant, as calcium- or magnesium-based salts can increase the concentration of the corresponding minerals in tofu. For example, Dimassi et al. [65] reported an ash content of 6.29% on a dry basis in chickpea tofu and associated the relatively high value with mineral uptake from the calcium coagulant used during processing. However, increased mineral content should not be interpreted as equivalent to increased bioavailability, particularly when phytate remains in the product. Auer et al. [3] demonstrated that the effects of processing on mineral bioavailability were closely related to changes in phytate content. Therefore, both mineral retention and the presence of compounds affecting mineral absorption should be considered when assessing the nutritional effects of tofu processing.

### 6.6. Process Scaling and Standardisation

Most available studies have been conducted under laboratory conditions, meaning that parameters considered optimal in small-scale experiments may not translate directly to industrial processing. This applies particularly to protein extraction, separation of insoluble fractions, heating, coagulant dosing and pressing, all of which may influence curd formation, water-holding capacity, yield and final product texture. Scale-up studies should therefore evaluate not only product quality but also process reproducibility, resource and energy requirements, equipment compatibility and the feasibility of maintaining consistent processing conditions at larger scales.

An additional area for future research is the utilisation of locally available legume species and cultivars. Findings for faba beans and different pea varieties demonstrate the potential of regional legumes as raw materials for tofu-type products [3]. However, differences in protein composition and technological behaviour among species and cultivars indicate that processing conditions should be adapted to the characteristics of the specific raw material rather than transferred directly from conventional soy tofu production. Future research should therefore focus on developing reproducible and scalable processing strategies that enable locally available legumes to be effectively utilised while maintaining desirable technological, nutritional and sensory properties.

## 7. Research Gaps and Prospects for Development

Despite growing interest in alternative plant protein sources, research on tofu produced from non-soy legumes remains fragmented. Relatively more information is available for chickpeas, peas, mung beans, faba beans and selected Vigna species, whereas evidence concerning the actual tofu production process remains limited for several other bean species and less commonly used legumes. Moreover, considerable methodological variation in raw-material preparation, protein extraction, heating, coagulation and product formation limits direct comparisons among studies. Future research should therefore prioritise comparative experiments conducted under standardised conditions rather than isolated demonstrations of the ability of individual raw materials to form tofu-type products [4,20].

A major research gap is the limited ability to predict suitable processing conditions from the characteristics of the raw material. Available evidence indicates that total protein content alone is insufficient to predict tofu-forming potential. Protein composition and functionality, extraction history and the presence of non-protein matrix components may all influence subsequent aggregation and gel formation [6,7,10,11]. Future studies should therefore move beyond the optimisation of individual variables towards integrated process design. In particular, coagulant concentration, acidification conditions, thermal treatment and the use of structure-modifying additives should be considered collectively. Studies on lentil and Bambara groundnut systems demonstrate that relatively small changes in additive concentration can substantially modify texture and gel-network organisation [43,52,58].

Emerging processing technologies provide additional opportunities to modify protein functionality before coagulation. Ultrasound, pulsed electric fields (PEFs), high-pressure homogenisation (HPH) and biological approaches such as fermentation have been investigated for their effects on protein extraction, structure and functionality [35,69,98,105]. For example, combined PEF and ultrasound treatment has been investigated for chickpea protein extraction, whereas ultrasound has been shown to enhance protein extraction from faba beans [68,71]. High-intensity ultrasonication of chickpea protein at 150 W for 30 min has also been reported to modify its physicochemical and structural characteristics and improve solubility, emulsifying capacity and foaming properties [106]. High-pressure homogenisation provides another approach for modifying the structural and functional properties of legume proteins [49]. However, much of this evidence originates from protein extraction or model protein systems rather than complete tofu production processes. The key research need is therefore not simply to demonstrate that these technologies modify proteins, but to establish whether such modifications improve subsequent coagulation, gel formation and final product quality in actual non-soy tofu systems.

Two important evidence gaps concern process scale-up and product stability. Although the need for reproducible scale-up has been recognised, validation of laboratory-optimised processing conditions at larger scales remains limited. Similarly, the available evidence is insufficient to establish general recommendations for the shelf life of non-soy tofu, particularly with regard to microbiological, physicochemical and sensory changes during refrigerated storage [59,64]. Future studies should therefore validate optimised processes under pilot- or industrially relevant conditions and establish product-specific storage stability and shelf-life criteria.

A further priority is the development of integrated criteria for evaluating product quality. Improvements in isolated outcomes such as fresh yield, hardness or protein concentration cannot be assumed to represent an overall improvement in the product. Comparative studies should therefore assess technological performance together with nutritional quality, antinutritional factors, mineral bioavailability and sensory acceptance using clearly defined and standardised analytical endpoints.

## 8. Conclusions

The available evidence demonstrates that the development of non-soy tofu cannot be approached as a simple substitution of soybean with another legume. The suitability of individual legumes depends on the interaction between protein composition and functionality, non-protein matrix components, and the processing conditions applied during extraction, heating and coagulation. Differences in storage-protein composition, protein solubility and thermal behaviour, together with the presence of starch, fibre and other matrix constituents, contribute to species-specific differences in curd formation, texture and water retention. Consequently, total protein content alone is not an adequate predictor of technological suitability.

The preparation method is a major determinant of the properties of non-soy tofu. Raw-material pre-treatment and extraction conditions influence protein recovery and the composition of the extract entering subsequent processing, while thermal treatment determines the extent of protein denaturation and aggregation before coagulation. Coagulant type and concentration, as well as acidification, enzymatic cross-linking and structure-modifying additives, further influence gel-network formation, texture and water retention. Available comparative evidence indicates that chickpea and faba bean can exhibit favourable curd-forming and textural properties under appropriate processing conditions, whereas pea, lentil and mung bean systems show particularly strong dependence on processing parameters. However, differences among experimental protocols currently prevent an unambiguous ranking of legume species.

Processing also affects nutritional properties and protein digestibility. Thermal treatment and coagulation can alter protein structure and accessibility to digestive enzymes, while fermentation and other processing steps may further modify digestibility and reduce selected antinutritional factors. Nevertheless, increased protein or mineral concentration in the final product does not necessarily correspond to improved nutritional bioavailability, particularly when phytate and other antinutritional compounds are retained. Therefore, evaluation of non-soy tofu should integrate protein digestibility and nutritional quality with technological performance. Sensory acceptance and safety are also important, as species-specific flavours, allergenicity, cross-reactivity and residual antinutritional or potentially toxic compounds may limit the suitability of otherwise technologically promising raw materials.

Overall, the evidence supports a raw-material-specific approach in which extraction, thermal treatment, coagulation and formulation are optimised as an integrated process. The major limitation preventing more definitive recommendations is the methodological heterogeneity of the available studies, including differences in raw-material preparation, protein concentration, coagulant dosage, pressing conditions and definitions of yield and water retention. Future research should therefore prioritise standardised comparative studies using complete tofu-type matrices to distinguish intrinsic species effects from processing effects and to establish reproducible technological and nutritional recommendations for non-soy tofu production.

## Figures and Tables

**Table 1:** Protein content and major protein fractions of selected legumes relevant to tofu-type product development.

Raw Material	Protein Content (% DM)	Major Protein Fractions	Characteristics of Major Protein Fractions	References
Chickpea (*Cicer arietinum*)	18–25	7S vicilin; 11S legumin; albumins	Globulins predominate, with both 7S and 11S fractions contributing substantially to the storage-protein fraction	[18,28,29]
Pea (*Pisum sativum*)	20–30	7S vicilin and convicilin; 11S legumin; albumins	Vicilin- and legumin-type globulins constitute the major storage-protein fractions, with their relative proportions varying among cultivars.	[18,30,31,32]
Faba bean (*Vicia faba*)	24–35	7S vicilin and convicilin; 11S legumin	Globulins constitute the predominant storage-protein fraction, with the relative proportions of vicilin- and legumin-type proteins varying among genotypes.	[33,34,35]
Lentil (*Lens culinaris*)	20–30	7S vicilin-type globulins; 11S legumin-type globulins; albumins	Globulins constitute the major storage-protein fraction, including vicilin- and legumin-type proteins.	[18,25,30]
Mung bean (*Vigna radiata*)	20–25	Predominantly 8S vicilin-type globulin; 11S globulin; albumins	An 8S vicilin-type globulin represents a major storage-protein fraction in mung bean.	[18,36,37]
Cowpea (*Vigna* *unguiculata*)	20–32	Globulins, albumins and glutelins	Globulins constitute the predominant storage-protein fraction	[38,39,40]
Bambara groundnut (*Vigna* *subterranea*)	17–25	Vicilin-type (7S) and legumin-type (11S) globulins; albumins	Storage proteins are predominantly globulin-type proteins, including vicilin- and legumin-related fractions	[41,42,43,44]
Lupin (*Lupinus* spp.)	30–45	α-, β-, γ- and δ-conglutins	Conglutins constitute the principal storage proteins, with β-conglutin representing a major vicilin-like fraction.	[18,22,23,45]

Explanatory notes: DM—dry matter. Values represent approximate ranges reported in the cited literature and may vary depending on species, cultivar, growing conditions and analytical methodology. Percentages describing protein fractions reported in individual studies may refer to different analytical bases and should therefore not be interpreted as directly interchangeable. The occurrence and concentration of bioactive and phytochemical compounds may also vary substantially depending on genotype, seed coat colour, growing conditions and processing. The compounds listed in the table represent major or commonly reported groups rather than fixed quantitative profiles. Source: Authors’ own compilation based on the cited literature.

**Table 2:** Compression of selected non-soy legumes in the context of tofu-type product development.

Raw Material	Relevant Technological Characteristics	Evidence from Tofu-Type Systems	Main Limitation	References
Chickpeas	Protein-rich matrix containing both 7S and 11S storage globulins and a substantial starch fraction	Favourable curd-forming and textural properties have been reported under selected processing conditions	Product properties remain dependent on extraction, coagulation conditions and the contribution of starch and other matrix components	[6,7,65]
Peas	Protein functionality is strongly dependent on extraction and processing conditions	Tofu-type products can be formed, but structural properties vary substantially with the processing protocol	Lower hardness has been reported under some standardised comparative conditions; process-specific optimisation is required	[9,11]
Faba Beans	Protein-rich, starch-containing matrix in which both protein and starch transformations contribute to structure formation	Compact gels and favourable water retention have been reported under selected processing conditions	Product properties depend on protein–starch interactions and processing conditions	[6,34,66]
Lentils	Proteins are capable of heat-induced aggregation and subsequent modification by coagulation or structure-modifying agents	CaSO_4_ and chitosan can substantially modify texture and network organisation	Product structure is highly sensitive to the coagulation and formulation conditions	[10,52]
Mung Beans	Protein functionality and starch-containing matrix respond strongly to thermal and coagulation conditions	Tofu-type products have been obtained, with properties strongly affected by water ratio, heating and coagulant concentration	High process dependence limits direct transfer of conditions between studies	[6,53,59]
Lupins	Protein profile is dominated by conglutins and differs from that of many other pulses	Evidence mainly concerns partial substitution of soybean with lupin rather than entirely lupin-based tofu	Limited evidence for soy-free, 100% lupin tofu systems	[62,63,67]
Bambara Groundnut	Protein–polysaccharide interactions can be modified through coagulant selection and hydrocolloid addition	Tofu-type products have been produced and their texture and sensory properties modified through formulation	Evidence remains limited to a relatively small number of studies and processing systems	[43,44]

Source: Authors’ own compilation based on the cited literature.

**Table 3:** Examples of processing conditions reported for non-soy tofu and tofu-type systems.

Raw Material	System and Processing Conditions	Selected Finding	References
Chickpeas	Protein-fraction system; CaSO_4_ or MgSO_4_; protein and coagulant concentrations varied	Product properties depended on both protein and coagulant concentration; selected CaSO_4_ with CaSO_4_ conditions producing distinct textural responses within the investigated system.	[6]
Lentils	Thermally gelled lentil system; CaSO_4_ (0–12 mM) or chitosan (0–1%) added before heating	CaSO_4_ and chitosan produced concentration-dependent changes in texture and microstructure; their effects differed according to the structuring agent and dosage.	[52]
Mung beans	Whole-bean tofu-type system; bean-to-water ratio, heating time and temperature, and CaSO_4_·2H_2_O concentration varied	Product structure and sensory properties were strongly affected by the combined processing conditions; among the conditions tested, a 1:5 bean-to-water ratio, heating at 90 °C for 15 min and 3% CaSO_4_·2H_2_O were selected as favourable.	[59]
Peas	Pea-based tofu-type system; processing conditions optimised within the study	A soy-free tofu-type product was obtained, but its properties were dependent on the processing conditions.	[9]
Bambara groundnut	Bambara groundnut tofu-type system; GDL, vinegar and hydrocolloid-containing formulations investigated	Coagulant conditions and hydrocolloid addition modified textural, rheological and sensory properties, demonstrating strong formulation dependence.	[43,44]
Faba beans	Whole-seed faba bean system; CaSO_4_ coagulation; raw-material-to-water ratio varied	Raw-material-to-water ratio and coagulation conditions affected the physicochemical and textural properties of the resulting tofu-type product.	[66]

Explanatory notes: CaSO_4_, calcium sulphate; MgSO_4_, magnesium sulphate; GDL, glucono-delta-lactone. Processing conditions are reported as described in the original studies and should not be directly compared across rows because the studies differed in raw-material form, protein concentration, processing sequence, concentration units and reference bases, and post-coagulation treatment. Source: Authors’ own compilation based on the cited literature.

**Table 4:** Reported relationships between selected processing or matrix factors and structural properties of non-soy tofu-type and model-gel systems.

Factor	Reported Structural Effect	Reported Effect on Measured Properties	References
Higher water retention	Greater retention of the aqueous phase within the gel network	May increase product moisture or water retention and can be associated with lower hardness, depending on the system	[6,66]
CaSO_4_/Ca^2+^ ions	Promotes protein aggregation and the formation of intermolecular associations	Can increase hardness and cohesiveness within an appropriate concentration range; the response is matrix- and concentration-dependent	[6,52]
Chitosan	Modifies protein aggregation and network organisation	Produces concentration-dependent changes in texture; excessive addition may result in a more porous, brittle and softer structure	[52,58]
mTGase	Promotes enzymatic protein cross-linking and can produce a denser network in model protein gels	Can increase WHC and modify mechanical properties in model-gel systems; validation in complete tofu matrices remains limited	[53,54]
Starch	Competes for water and interacts with the protein network during heating and gel formation	May modify network continuity, water retention and texture depending on starch content and thermal conditions	[34,65]
Type of raw material	Species-specific protein composition and non-protein matrix components influence aggregate and pore organisation	Contributes to differences in texture and other product properties, although processing conditions may confound direct species comparisons	[6,7,60]

Explanatory notes: CaSO_4_, calcium sulphate; Ca^2+^, calcium ions; mTGase, microbial transglutaminase; WHC, water-holding capacity. The reported relationships are system-dependent and should not be interpreted as universal effects, because the cited studies differ in raw-material form, protein concentration, processing conditions and experimental design; some evidence is derived from model protein-gel systems rather than complete tofu-type matrices. Source: Authors’ own compilation based on the cited literature.

## Data Availability

No new data were created or analysed in this study. Data sharing is not applicable to this article.
